# Supplementary material for: Surgical vs. transcatheter aortic valve replacement in patients over 75 years with aortic stenosis: sociodemographic profile, clinical characteristics, quality of life and functionality
Source: PeerJ. 2023 Sep 20;11:e16102. doi: 10.7717/peerj.16102 (PMC10538279; doi:10.7717/peerj.16102)
Supplement: Supplemental Information 1 [file peerj-11-16102-s001.docx]

S1_Table. Evolution of quality of life according to the SF-36 domains atbaseline, 1 month, 6 months and 1year follow-up after the intervention.

|  | **Baseline** | | **1 month** | | **6 months** | | **1 year** | |
| --- | --- | --- | --- | --- | --- | --- | --- | --- |
|  | **TAVR**  **n=113** | **SAVR**  **n=113** | **TAVR**  **n=106** | **SAVR**  **n=106** | **TAVR**  **n=103** | **SAVR**  **n=105** | **TAVR**  **n=98** | **SAVR**  **n=103** |
| **SF-36 GLOBAL** | 46.6  (IQR 32-60)  (SD 19.8)  Median=48 | 57.8  (IQR 45.4- 71.4)  (SD 17.9)  Median=59.2 | 62.4  (IQR 45.3-81)  (SD 23.3)  Median=66.8 | 58.4  (IQR 43.9-74.9)  (SD 20.5)  Median=61.1 | 66  (IQR 53 -85)  (SD 23.1)  Median=71 | 76.4  (IQR 68.4-88)  (SD 16.1)  Median=81.7 | 67.6  (IQR 49-86.7)  (SD 22.6)  Median=74.5 | 79.4  (IQR 72.8-91.1)  (SD 16.6)  .  Median=85.2 |
| *p* value | **<0.01** | | 0.11 | | **<0.01** | | **<0.01** | |
| **Physical Function** | 35.9  (IQR 20-55)  (SD 22.8)  Median=35 | 57.5  (IQR 40-75)  (SD 23.9)  Median=60 | 52.9  (IQR 30-75)  (SD 25.9)  Median=55 | 53  (IQR 35-75)  (SD 26.6)  Median=55 | 58.5  (IQR 40-80)  (SD 27.3)  Median=60 | 72.8  (IQR 65-90)  (SD 20.8)  Median=80 | 60.1  (IQR 40-85)  (SD 27.8)  Median=60 | 77.7  (IQR 65-95)  (SD 19.2)  Median=85 |
| *p* value | **<0.01** | | 0.97 | | **<0.01** | | **<0.01** | |
| **Physical Role** | 29.6  (IQR 0-75)  (SD 43.6)  Median=0 | 32.6  (IQR 0-75)  (SD 43)  Median=0 | 45.8  (IQR 0-100)  (SD 48)  Median=25 | 19.1  (IQR 0-25)  (SD 36.3)  Median=0 | 63.2  (IQR 0-100)  (SD 47)  Median=100 | 62.1  (IQR 0-100)  (SD 46.1)  Median=100 | 61  (IQR 0-100)  (SD 47.8)  Median=100 | 65.5  (IQR 0-100)  (SD 45.3)  Median=100 |
| *p* value | 0.47 | | **<0.01** | | 0.84 | | 0.62 | |
| **Bodily Pain** | 62.4  (IQR 32-100)  (SD 34.4)  Median=70 | 73.6  (IQR 57.5-100)  (SD 26.9)  Median=70 | 66.6  (IQR 32.5-100)  (SD 34.9)  Median=80 | 73.6  (IQR 57.5-100)  (SD 29)  Median=80 | 65.2  (IQR 22.5-100)  (SD 37.1)  Median=80 | 77.4  (IQR 70-100)  (SD 29.2)  Median=90 | 74.2  (IQR 52-100)  (SD 30.1)  Median=80 | 79.6  (IQR 67.5-100)  (SD 27.7)  Median=100 |
| *p* value | **0.02** | | 0.43 | | 0.12 | | 0.22 | |
| **General Health** | 54.2  (IQR 40-70)  (SD 19.5)  Median=55 | 60.4  (IQR 45-75)  (SD 18.5)  Median=60 | 65.6  (IQR 55-85)  (SD 22.1)  Median=70 | 65  (IQR 55-80)  (SD 17.8)  Median=65 | 63.6  (IQR 45-80)  (SD 23.2)  Median=70 | 70.6  (IQR 60-85)  (SD 19.2)  Median=75 | 63.9  (IQR 50-80)  (SD 23.1)  Median=65 | 72.4  (IQR 55-90)  (SD 20.3)  Median=80 |
| *p* value | **0.03** | | 0.35 | | **0.04** | | 0.07 | |
| **Vitality** | 40.9  (IQR 20-60)  (SD 26.3)  Median=40 | 48.8  (IQR 25-70)  (SD 27)  Median=50 | 52.9  (IQR 40-75)  (SD 27.8)  Median=55 | 46.8  (IQR 30-65)  (SD 26.2)  Median=45 | 57.5  (IQR 40-80)  (SD 27.9)  Median=60 | 68.2  (IQR 50-90)  (SD 25.9)  Median=75 | 58  (IQR 40-80)  (SD 26.2)  Median=60 | 69.4  (IQR 55-90)  (SD 25.8)  Median=75 |
| *p* value | **0.03** | | **0.047** | | **0.04** | | **0.01** | |
| **Social Function** | 59.8  (IQR 23-100)  (SD 38.5)  Median=65 | 79.2  (IQR 67.5-100)  (SD 28.4)  Median=90 | 77.8  (IQR 62.5-100)  (SD 36.1)  Median=100 | 75.5  (IQR 55-100)  (SD 34.2)  Median=100 | 83.4  (IQR 87.5-100)  (SD 32)  Median=100 | 91.7  (IQR 100-100)  (SD 19.4)  Median=100 | 82  (IQR 77.50-100)  (SD 30.7)  Median=100 | 95.9  (IQR 100-100)  (SD 14.8)  Median=100 |
| *p* value | **<0.01** | | 0.22 | | 0.16 | | **<0.01** | |
| **Emotional Role** | 44.5  (IQR 0-100)  (SD 47.7)  Median=0 | 66.4  (IQR 0-100)  (SD 44.9)  Median=100 | 62.9  (IQR 0-100)  (SD 46.5)  Median=100 | 55.7  (IQR 0-100)  (SD 48.4)  Median=100 | 67  (IQR 0-100)  (SD 45.9)  Median=100 | 86.3  (IQR 100-100)  (SD 33.6)  Median=100 | 68  (IQR 0-100)  (SD 45.9)  Median=100 | 88.7  (IQR 100-100)  (SD 31.5)  Median=100 |
| *p* value | **<0.01** | | 0.26 | | **<0.01** | | **<0.01** | |
| **Mental Health** | 63  (IQR 40-88)  (SD 27.3)  Median=64 | 73.3  (IQR 60-92)  (SD 22.6)  Median=80 | 71.8  (IQR 52-96)  (SD 27)  Median=80 | 73.3  (IQR 56-96)  (SD 27.7)  Median=84 | 71.2  (IQR 56-100)  (SD 28.2)  Median=76 | 86.4  (IQR 80-100)  (SD 15.9)  Median=92 | 71.3  (IQR 60-96)  (SD 25.4)  Median=76 | 86.6  (IQR 80-100)  (SD 16.4)  Median=92 |
| *p* value | **<0.01** | | 0.72 | | **<0.01** | | **<0.01** | |
| **Health Transition** | 29.4  (IQR 25-50)  (SD 24.6)  Median=25 | 29.5  (IQR 25-50)  (SD 21.9)  Median=25 | 64.4  (IQR 50-75)  (SD 26.2)  Median=75 | 57.6  (IQR 25-75)  (SD 28.2)  Median=75 | 64  (IQR 50-75)  (SD 28.8)  Median=75 | 71.4  (IQR 50-100)  (SD 26.7)  Median=75 | 70.2  (IQR 50-100)  (SD 25.3)  Median=75 | 79.1  (IQR 75-100)  (SD 23.2)  Median=75 |
| *p* value | 0.62 | | 0.07 | | **0.06** | | **<0.01** | |

IQR: interquartile Range; SD: Standard Deviation; TAVR: transcatheter aortic valve replacement; SAVR: surgical aortic valve replacement; SF-36: The 36-Item Short Form Health Survey questionnaire. *p* value: Wilcoxon test.
