# Supplementary material for: Surgical vs. transcatheter aortic valve replacement in patients over 75 years with aortic stenosis: sociodemographic profile, clinical characteristics, quality of life and functionality
Source: PeerJ. 2023 Sep 20;11:e16102. doi: 10.7717/peerj.16102 (PMC10538279; doi:10.7717/peerj.16102)
Supplement: Supplemental Information 2 [file peerj-11-16102-s002.docx]

S2_Table. Evolution of quality of life according to the EuroQol-5D domains at one-year follow-up after the intervention.

|  | **Baseline** | | | **1 month** | | **6 months** | | **1 year** | |
| --- | --- | --- | --- | --- | --- | --- | --- | --- | --- |
|  | **TAVR**  **n=113** | **SAVR**  **n=113** | | **TAVR**  **n=106** | **SAVR**  **n=106** | **TAVR**  **n=103** | **SAVR**  **n=105** | **TAVR**  **n=98** | **SAVR**  **n=103** |
| **EuroQol-5D GLOBAL** | 53.9 | 62.5 | | 61.7 | 64.6 | 66.  7 | 75.4 | 68.1 | 76.4 |
|  | (IQR 50-70) | (IQR 50 -75) | | (IQR 50-80) | (IQR 50 -80) | (IQR 50-80) | (IQR 70-90) | (IQR 50-80) | (IQR 70-90) |
|  | (SD 18.2) | (SD 15.5) | | (SD 20.8) | (SD 21.4) | (SD 20.2) | (SD 17.5) | (SD 21.4) | (SD 18.8) |
|  | Range=100 | Range=75 | | Range=90 | Range=100 | Range=90 | Range=80 | Range=95 | Range=90 |
|  | Median=50 | Median=60 | | Median=60 | Median=70 | Median=70 | Median=80 | Median=70 | Median=80 |
| *p* value | **<0.01** | | | 0.32 | | **< 0.01** | | **< 0.01** | |
| **Mobility** |  | | |  | |  | |  | |
| I have no problems | 66 (58%) | | 85 (75%) | 78 (74%) | 77 (73%) | 73 (71%) | 97 (92%) | 71 (72%) | 93 (90%) |
| I have some problems | 44 (39%) | | 28 (25%) | 27 (25%) | 27 (25%) | 29 (28%) | 8 (8%) | 26 (27%) | 10 (10%) |
| Confined to bed | 3 (3%) | | 0 | 1 (1%) | 2 (2%) | 1 (1%) | 0 | 1 (1%) | 0 |
| *p* value | **< 0.01** | | | 0.84 | | **< 0.01** | | **< 0.01** | |
| **Self Care** |  | | |  | |  | |  | |
| I have no problems | 84 (74%) | | 105 (93%) | 84 (79%) | 74 (70%) | 79 (77%) | 94 (90%) | 74 (76%) | 97 (94%) |
| I have some problems washing or dressing myself | 21 (19%) | | 4 (4%) | 11 (10%) | 21 (20%) | 12 (12%) | 9 (9%) | 17 (17%) | 4 (4%) |
| I am unable to wash or dress myself | 8 (7%) | | 4 (4%) | 11 (10%) | 11 (10%) | 12 (12%) | 2 (2%) | 7 (7%) | 2 (2%) |
| *p* value | **< 0.01** | | | 0.15 | | **0.01** | | **< 0.01** | |
| **Usual Activities** |  | | |  | |  | |  | |
| I have no problems | 53 (47%) | | 77 (68%) | 55 (52%) | 36 (34%) | 55 (53%) | 79 (75%) | 60 (61%) | 84 (82%) |
| I have some problems | 40 (35%) | | 28 (25%) | 27 (25%) | 44 (42%) | 23 (22%) | 19 (18%) | 24 (24%) | 14 (14%) |
| I am unable to perform my usual activities | 20 (18%) | | 8 (7%) | 24 (23%) | 26 (25%) | 25 (24%) | 7 (7%) | 14 (14%) | 5 (5%) |
| *p* value | **< 0.01** | | | **0.02** | | **<0.01** | | **<0.01** | |
| **Pain/discomfort** |  | | |  | |  | |  | |
| None | 52 (46%) | | 57 (50%) | 59 (56%) | 71 (67%) | 57 (55%) | 72 (69%) | 60 (61%) | 71 (69%) |
| Moderate | 42 (37%) | | 45 (40%) | 22 (21%) | 26 (25%) | 23 (22%) | 19 (18%) | 18 (18%) | 18 (17%) |
| Extreme | 19 (17%) | | 11 (10%) | 25 (24%) | 9 (8%) | 23 (22%) | 14 (13%) | 20 (20%) | 14 (14%) |
| *p* value | 0.29 | | | **0.01** | | 0.12 | | 0.39 | |
| **Anxiety/depression** |  | | |  | |  | |  | |
| I am not anxious or depressed | 61 (54%) | | 77 (68%) | 83 (78%) | 81 (76%) | 78 (76%) | 95 (90%) | 75 (77%) | 92 (89%) |
| I am moderately anxious or depressed | 38 (34%) | | 34 (30%) | 18 (17%) | 20 (19%) | 18 (17%) | 10 (10%) | 17 (17%) | 9 (9%) |
| I am extremely anxious or depressed | 14 (12%) | | 2 (2%) | 5 (5%) | 5 (5%) | 7 (7%) | 0 | 6 (6%) | 2 (2%) |
| *p* value | **< 0.01** | | | 0.94 | | **< 0.01** | | **0.04** | |

IQR: Interquartile Range; SD: Standar Deviation; TAVR: transcatheter aortic valve replacement; SAVR: surgical aortic valve replacement, EuroQol-5D: EuroQol five-dimension scale. *p* value: Pearson's Chi-Squared Test
