# Supplementary material for: Surgical vs. transcatheter aortic valve replacement in patients over 75 years with aortic stenosis: sociodemographic profile, clinical characteristics, quality of life and functionality
Source: PeerJ. 2023 Sep 20;11:e16102. doi: 10.7717/peerj.16102 (PMC10538279; doi:10.7717/peerj.16102)
Supplement: Supplemental Information 4 [file peerj-11-16102-s004.docx]

| VARIABLE | LEGENDS | CODE |
| --- | --- | --- |
| CENTER | Hospital Universitario Marqués de Valdecilla HUMV; Hospital Universitario de Salamanca HUSA | HUMV; HUSA |
| PROCEDURE | SAVR: Surgical Aortic Valve Replacement; TAVR: Transcatheter Aortic Valve Replacement | SAVR; TAVR |
| GENDER |  | 1:MALE  2:FEMALE |
| CIVIL STATUS |  | 1:Married  2:Single  3:Widowed  4:Separated/divorced |
| Who do you live with? |  | 1:Alone  2:Family  3:Social-health care institution |
| STUDIES | Level 1: primary education or 5 years of EGB or equivalent; 2nd grade: Elementary Baccalaureate, School Graduate, complete EGB, Level 1 and 2 Vocational Training, Higher Baccalaureate; Level 3: Higher Technical School Degrees, University School Graduates and University College or University College Graduates, Bachelor's Degrees. | 1:Illiterate  2:No education  3:Level 1  4:Level 2  5:Level 3 |
| SMOKING |  | 0: NO  1: SI |
| HBP | HBP: High Blood Pressure | 0: NO  1: SI |
| DM | DM: Diabetes Mellitus | 0: NO  1: SI |
| DLP | DLP: Dyslipemia | 0: NO  1: SI |
| PRIOR STROKE |  | 0: NO  1: SI |
| TIA | TIA: Transient Ischemic Attack | 0: NO  1: SI |
| Peripheral Arteriopathy |  | 0: NO  1: SI |
| PRIOR MI | MI: Acute Myocardial Infarction | 0: NO  1: SI |
| PRIOR PTCA | PTCA: Percutaneous Transluminal Coronary Angioplasty | 0: NO  1: SI |
| PRIOR CABG | CABG: Coronary artery bypass grafting | 0: NO  1: SI |
| PRIOR SAVR | SAVR: Surgical Aortic Valve Replacement | 0: NO  1: SI |
| Prior MVR | MVR: Mitral Valve Replacement | 0: NO  1: SI |
| Other Prior Cardiac Surgery |  | 0: NO  1: SI |
| Prior pacemaker |  | 0: NO  1: SI |
| Prior ICD | ICD: implantable cardioverter-defibrillator | 0: NO  1: SI |
| PRIOR AF | AF: Atrial fibrillation | 0: NO  1: SI |
| PRIOR ANTICOAGULATION |  | 0: NO  1: SI |
| PRIOR COPD | COPD: Chronic Obstructive Pulmonary Disease; | 0: NO  1: SI |
| Anxiety-Depression Syndrome |  | 0: NO  1: SI |
| Cognitive Impairment |  | 0: NO  1: SI |
| Prior liver disease |  | 0: NO  1: SI |
| SYMPTOMS |  | 1:Dyspnea  2:Angina  3:Syncope  4: Dyspnea + Angina |
| NYHA | NYHA: New York Heart Association | 1:Class I  2:Class II  3:Class III  4:Class IV |
| PRIOR HB | Hb: Hemoglobin |  |
| PRIOR GF | GF: Glomerular Filtration |  |
| PRIOR LVEF | LVEF: Left Ventricular Ejection Fraction |  |
| SF36 GLOBAL | BASELINE; 1 MONTHS; 6 MONTHS; 12 MONTHS |  |
| Physical Function |  |  |
| Physical Role |  |  |
| Bodily Pain |  |  |
| General Health |  |  |
| Vitality |  |  |
| Social Function |  |  |
| Emotional Role |  |  |
| Mental Health |  |  |
| Health Transition |  |  |
| EuroQol-5D GLOBAL BASELINE |  |  |
| Mobility |  |  |
| Self care |  |  |
| Usual Activites |  |  |
| Pain/Discomfort |  |  |
| Anxiety/depression |  |  |
| BARTHEL SCALE |  |  |
